# Recurrent mucinous carcinoma with sarcomatoid and sarcomatous mural nodules: a case report and literature review

**DOI:** 10.3389/fonc.2024.1387700

**Published:** 2024-06-06

**Authors:** Simin Li, Jingyu Zhu, Na Jiang, Yanping Guo, Meng Hou, Xi Liu, Jin Yang, Xiaofeng Yang

**Affiliations:** ^1^ Department of Gynecology and Obstetrics, First Affiliated Hospital of Xi ‘an Jiaotong University, Xi’an Jiaotong University, Xi’an, Shaanxi, China; ^2^ Department of Pathology, First Affiliated Hospital of Xi ‘an Jiaotong University, Xi’an Jiaotong University, Xi’an, Shaanxi, China; ^3^ Department of Oncology, First Affiliated Hospital of Xi ‘an Jiaotong University, Xi’an Jiaotong University, Xi’an, Shaanxi, China

**Keywords:** mucinous ovarian carcinoma, sarcomatous mural nodules, sarcoma-like mural nodule, undifferentiated sarcoma, KRAS, TP53, mutation

## Abstract

Ovarian mucinous tumors with sarcomatous mural nodules are rare. Sarcomatous nodules have a bad prognosis. Its diagnosis and treatment are controversial.It is still controversial whether malignant mural nodules represent a dedifferentiated form of mucinous tumors or collisional tumors. This is a case report of a 32-year-old female diagnosed with ovarian mucinous tumor recurred as a mucinous carcinoma combined with sarcomatoid and undifferentiated sarcoma mural nodules after surgery and chemotherapy. The primary lesion did not have a sarcomatous component after comprehensive sampling and repeated review, while the recurrent lesion had a predominantly sarcomatous component. The patient received a second operation and postoperative chemotherapy plus Anlotinib with no progression at 16 months of follow-up. Primary mucinous carcinoma and sarcomatous mural nodules revealed the same K-RAS mutation(c.35G>T, pG12V), TP53 mutation (c.817C>T, p.R273C), MLL2 mutation(c.13450C>T, p.R4484) and NF1 mutation(c.7876A>G, p.S2626G). We present a comprehensive analysis on morphologic characteristics, molecular detection results, clinical management, and prognosis of ovarian mucinous tumors with mural nodules of sarcomatoid and undifferentiated sarcoma. Mutation sharing between primary mucinous carcinoma and recurrent sarcomatous nodules supports monoclonal origin of primary and recurrent tumors, suggesting a tendency for sarcomatous differentiation during the progression of epithelial tumors. Malignant mural nodules represent dedifferentiation in mucinous ovarian tumors rather than collision of two different tumor types. Therefore, it is imperative to conduct comprehensive sampling, rigorous clinical examination, and postoperative follow-up in order to thoroughly evaluate all mural nodules of ovarian mucinous tumors due to their potential for malignancy and sarcomatous differentiation.

## Introduction

Ovarian mucinous tumors with mural nodules are a special type of ovarian surface epithelial-stromal tumor. Ovarian mucinous tumors, whether benign, borderline, or malignant, may be associated with a variety of mural nodules, including sarcomatoid mural nodules (SLMNs), sarcoma, and anaplastic carcinoma. Only approximately 12 cases of sarcomatous nodules have been reported in the last 30 years. Reviewing the literature, these nodules range from benign histology, such as sarcomatoid, to malignant histology, including anaplastic carcinoma, carcinosarcoma, and sarcoma (fibrosarcoma, rhabdomyosarcoma, osteosarcoma, and pleomorphic undifferentiated sarcoma) (**Table 1**). Among these, SLMNs are the most common and have a relatively good prognosis, while sarcomatous nodules are the rarest and are considered to have the worst prognosis (11). To the best of our knowledge, there are no reports of recurrent mucinous carcinoma with mural nodules of ovarian mucinous cystadenocarcinoma. Due to its rarity, most of the knowledge comes from case reports in the literature. Their pathological diagnosis and treatment are still inconclusive due to few reports. The histogenesis of the mural nodule remains unknown.

**Table 1:** Summary of the cases of ovarian mucinous tumors with malignant mural nodules.

	case	age of patients	pathology diagnosis	initial symptom	mural nodule	FIGO stage	therapy	follow-up
**Jaime Prat and Robert E. Scully(1979) (** 1)	2(Case 1)	61	mucious cystadenoma	abdominal swelling	fibrosarcoma	Unkown	TAH+BSO+appen+RT	36months(hepatic metastases)
**Jaime Prat and Robert E. Scully(1979) (** 1)	2(Case 2)	49	mucinous cystadenocarcinoma	Progressive abdominal swelling 1month	undifferentiated sarcoma	Unkown	BSO+omen+PALNB	1week(renal failure)
**Jan A,Bruijin et al(1987) (** 2)	1	27	mucinous cystadenocarcinoma	lower abdominal swelling	fibrosarcoma	Ia	USO(left)+omen	Unkown
**Takashi Tsujimura and Kiyoshi Kawano(1992) (** 3)	1	57	mucinous cystadenocarcinoma	lower abdominal mass	Rhabdomyosarcoma	Unkown	TAH+BSO+CHT	NED,3 months
**M. A. RAHILLY(1994) (** 4)	1	69	cystadenocarcinoma	lower abdominal pain	fibrosarcoma	II	TAH+BSO+appendicectomy+CHT	NED, 12months
**Mohamed et al(2014) (** 5)	1	25	mucious carcinoma	abdominal swelling with ascites	highly malignant sarcoma	Ia	excision of the ovarian mass and complete staged surgery	Unkown
**Marie McFarland et al(2015) (** 6)	2(Case 1)	34	mucinous carcinoma of intestinal type	persistent vaginal bleeding and abdominal swelling	Osteosarcoma	Ia	TAH+BSO	NED,18months
**Marie McFarland et al(2015) (** 6)	2(Case 2)	18	borderline mucinous tumor of intestinal type	abdominal swelling	Osteosarcoma	Ic	left oophorectomy+omen	NED,12month
**Yan Zhang et al(2017) (** 7)	2(Case1)	60	mucinous carcinoma and cystadenoma and borderline malignancy cystadenoma	Lower abdominal pain	sarcomatous	I	BSO+omen	Unkown
**Wen Wang et al(2017) (** 8)	1	42	mucinous borderline tumor		low-grade endometrial stromal sarcoma	II	left salpingo-oophorectomy and adhesiolysis	NED,10months
**Ying Shao et al(2020) (** 9)	3(Case 2)	41	mucious carcinoma	pelvic mass	sarcoma	IIa	TAH+BSO+omen+ PPLND+CHT	18months(lung metastasis); NED,40months
**Ling-Hui Chu et al(2021) (** 10)	1	65	mucious cystadenoma	Progressive abdominal swelling and inadequate uptake	osteosarcoma	Ic	USO(right)	NED,16months

TAH, total abdominal hysterectomy; BSO, bilateral salpingo-oophorectomy; USO, unilateral salpingo-oophorectomy; appen, appendectomy; omen, omentectomy; CHT, chemotherapy; DOD, died of disease; NED, no evidence of disease; RT, radiation therapy; PALNB, para-aortic lymph node biopsy; PPLND, pelvic/para-aortic lymph node dissection.

Using immunohistochemistry and a 1021 gene panel of next-generation gene sequencing for tumors before and after recurrence, we investigated a case of primary ovarian mucinous cystadenocarcinoma and recurrence as mucinous carcinoma with SLMNs and undifferentiated sarcoma. Four common mutations were found in the two groups. This study retrospectively analyzed the clinical manifestation, treatment, prognosis, pathology, immunophenotype and genetic mutations in primary and recurrent tumors to elucidate their classification, origin and treatment. We also summarize similar cases to raise awareness of this disease among clinicians.

## Case presentation

A 33-year-old female patient presented with a 10-day history of frequent urination and was subsequently referred to First Affiliated Hospital of Xi ‘an Jiaotong University in July 2021. Gynecological examination revealed a well-defined pelvic mass measuring 15 × 15 × 14 cm on the right side. The ultrasound demonstrated a cystic mass measuring 15.2 × 9.6 ×13.7 cm adjacent to the uterus on the left side. Laboratory investigations revealed elevated levels of carcinoembryonic antigen (CEA) at 290 ng/ml, carbohydrate antigen (CA199) exceeding 1000 U/ml, carbohydrate antigen (CA125) at 583 U/ml, and carbohydrate antigen (CA724) surpassing 300 U/ml. Pelvic magnetic resonance imaging (MRI) exhibited a large cystic and solid mass in the left ovary with evidence of minimal intracystic hemorrhage. Computed tomography (CT) scan indicated cystic and solid ovarian neoplasms along with minimal fluid accumulation within the abdomen and pelvis. She underwent appendectomy due to appendicitis in 2004.The patient’s family history revealed her mother died of ovarian cancer.

The patient underwent a total abdominal hysterectomy, bilateral salpingo-oophorectomy, and omentectomy at the First Affiliated Hospital of Xi’an Jiaotong University on August 4, 2021. Intraoperatively, a left-sided cystic and solid ovarian mass measuring approximately 18 × 10 × 10 cm with a smooth surface and intact capsule was observed. Intraoperative frozen section pathology revealed a moderately differentiated adenocarcinoma of the left ovary. The final diagnosis was stage Ia ovarian mucinous cystadenocarcinoma according to FIGO classification. Post-surgery, the patient received intraperitoneal infusion chemotherapy with cisplatin and systemic intravenous chemotherapy with paclitaxel and carboplatin.

After completing four courses of chemotherapy, an MRI scan conducted four months post-surgery detected a metastasis in the lower abdomen measuring 5 × 4.8 × 4 cm. A biopsy of the mass confirmed its mucinous nature with sarcomatoid nodules. Pathology consultation at the Cancer Prevention and Treatment Centre of Sun Yat-sen University indicated that mucinous tumors with sarcomatoid nodules and tend to metastatic lesions.

**Figure 1:**
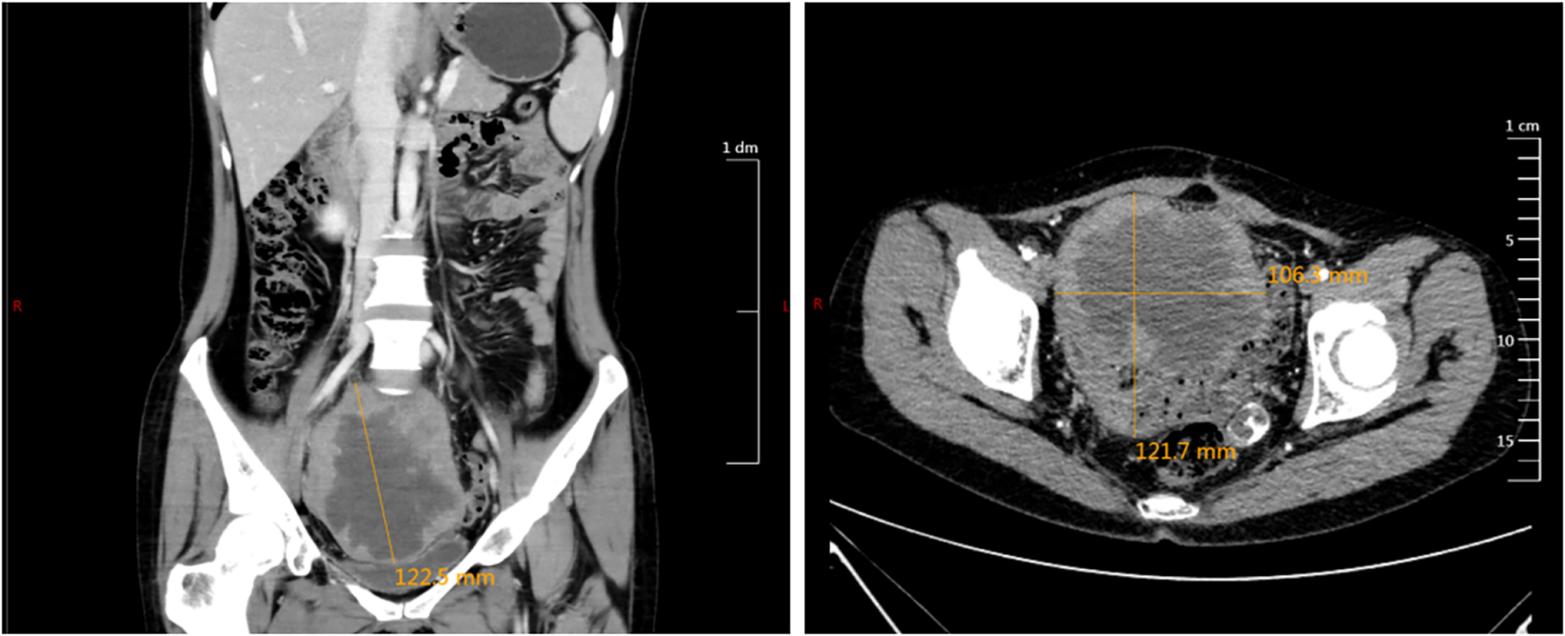
CT scan prior to the second operation. Hepatogastric space, mesenteric space, the largest of multiple soft tissue nodules in front of the right psoas major muscle at the level of the fifth lumbar vertebra, on the left side of the pelvic floor was approximately 13.6*11*9.4 cm in size, which was uneven and partially accompanied by calcified density.

To ascertain the recurrence status and presence of distant metastases, positron emission tomography/computed tomography (PET/CT) was conducted on March 1, 2022. The imaging revealed multiple soft tissue nodules in the splenogastric space, mesenteric space, anterior to the right psoas major muscle at the level of lumbar vertebra 5, pelvis, and left side of the pelvic floor. Some nodules exhibited calcified density and increased glucose metabolism, indicating metastatic involvement. Tumor markers were approximately normal. Considering its rapidly growing biological behavior and pathology findings, a potential recurrence of mucinous tumor with sarcomatous component was considered. The patient underwent two cycles of chemotherapy (doxorubicin and cisplatin) combined with bevacizumab. Subsequent CT scans showed no significant changes. Solid margins were observed along with central necrosis within the pelvic mass (previously exhibiting solid center with calcified spots) (**Figure 1**). These observations suggest minimal disease progression and limited response to chemotherapy. Therefore, surgical intervention was proposed.

The secondary tumor cell reduction surgery was performed on May 10, 2022. Intraoperatively, a solid mass measuring 16 cm x 11 cm x 9 cm was observed in the pelvic cavity, adhering to the greater omentum, peritoneum, small intestine, and Douglas pouch. Two metastatic lesions measuring approximately 3 cm and 2 cm in diameter were identified in the mesentery of the right paracolic sulci. They exhibited hardness and adherence to both colon and peritoneum. Additionally, two metastatic lesions with an approximate diameter of 2 cm each were found in the splenic flexure. Multiple metastatic foci were observed on the surface of the small intestine’s mesentery. Intraoperative frozen section pathology revealed epithelioid malignant tumors with giant cells with necrosis. All macroscopically visible lesions were successfully excised during surgery. Postoperatively, hyperthermic intraperitoneal chemotherapy (HIPEC) was administered along with six cycles of ifosfamide plus doxorubicin chemotherapy regimen. Subsequently, the patient received anlotinib maintenance therapy. Regular follow-up for a period of sixteen months without any signs of disease progression.

## Pathologic results

### Primary tumor

Grossly, the left ovarian cystic mass measured 15.5 × 13 × 4 cm and exhibited a grayish red color with a smooth surface. Cross-sections revealed both cystic and solid areas. The multilocular cystic regions contained dark brown and bloody fluid ranging in diameter from 0.5-5 cm. The solid area measured 6 cm × 6 cm and displayed a grayish white section.

**Figure 2:**
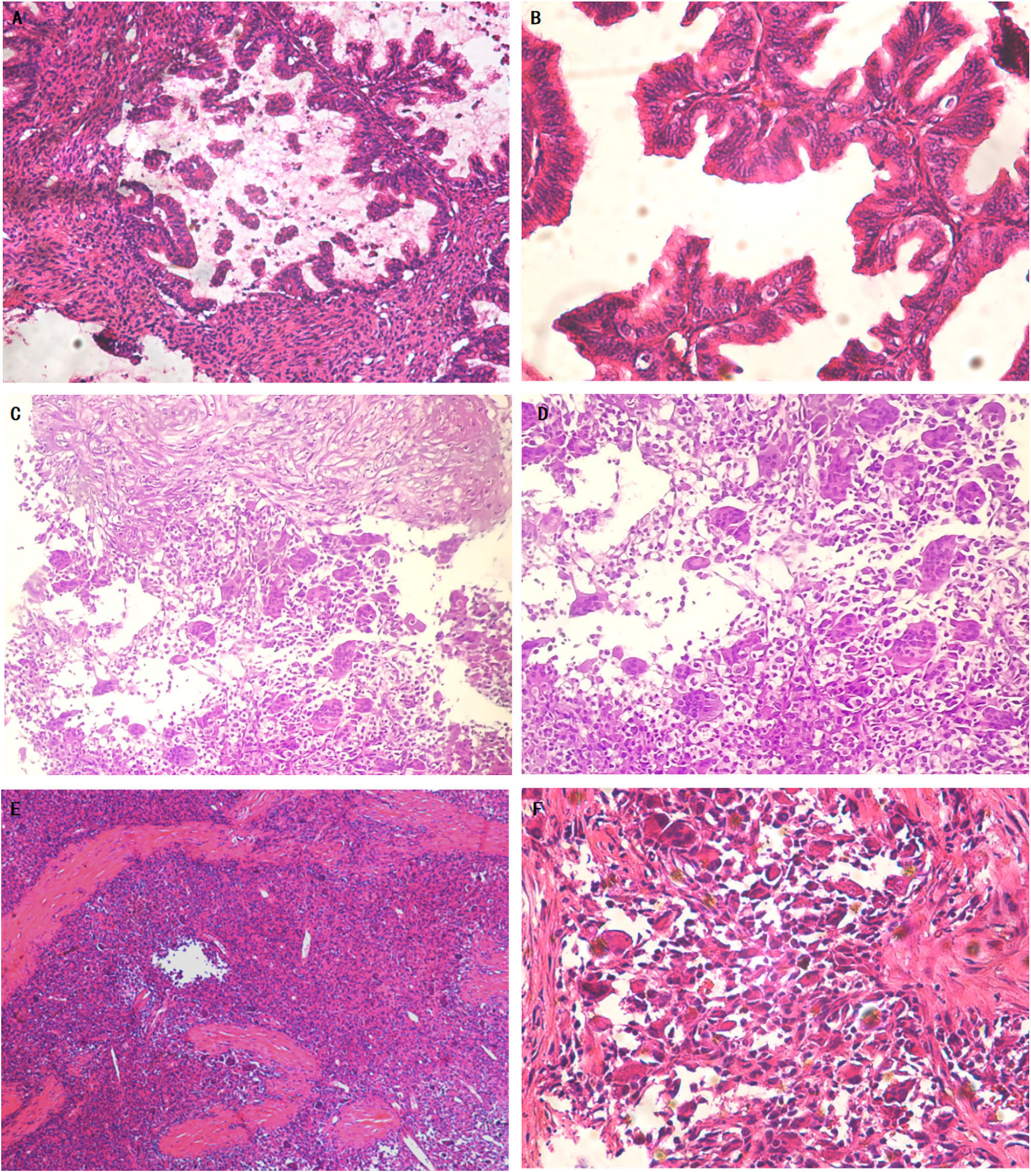
Primary mucinous cystadenocarcinoma of the ovary and recurrent mucinous carcinoma with sarcomatoid mural nodule and local undifferentiated sarcoma (Hematoxylin and eosin). Representative microscopic photos demonstrate the well-differentiated to moderately differentiated, invasive mucinous carcinoma **(A)**. On higher power, some of the glands showed back-to-back arrangement. The tumor cells were highly columnar with abundant cytoplasm, most of which was mucilage **(B)**. The pathology of the mass biopsy showed a recurrent mucinous tumor with sarcomatoid nodules. Focal mucinous carcinoma with abundant cytoplasm, most of which was mucinous, is seen in the upper part. In the lower part there are sarcomatoid nodules with multinucleated giant cells **(C)**. On higher power, the sarcomatoid nodules are composed of sarcomatoid multinucleated cells and multinucleated giant cells **(D)**. Sarcomatoid mural nodule with local undifferentiated sarcoma **(E)**. On higher power, multinucleated tumor giant cells were scattered or scaly. The tumor cells showed pleomorphic changes arranged in no fixed direction **(F)**.

Microscopically, the tumor tissue demonstrated an adenoidal arrangement, with some glands exhibiting a back-to-back arrangement. Within the glandular cavity, tumor cells formed complex papillary structures, accompanied by visible fibrovascular axes at the center of each papilla (**Figure 2A**). These tall columnar tumor cells possessed abundant cytoplasm, primarily mucilaginous. Part of the cytoplasm appeared eosinophilic. The nuclei were rod-shaped or oval with distinct nuclear membranes, prominent nucleoli, and evident nuclear division (**Figure 2B**).

The final diagnosis indicated highly to moderately differentiated papillary mucinous cystadenocarcinoma of the left ovary without extension to the surface of the left ovary. Subsequently, the patient was referred to the Cancer Prevention and Treatment Centre of Sun Yat-sen University for pathological consultation which confirmed the initial diagnosis: microscopic morphology consistent with well to moderately differentiated papillary mucinous cystadenocarcinoma of the ovary.

Immunohistochemical analysis revealed strong positive expression of CAM52 and Pax-8 in tumor cells. Tumor cells expressed Ki-67 at approximately 60%, while SATB2, H3.3G34 W, WT1,and p63 exhibited negative expression.

### Recurrence tumor

Macroscopically, the pelvic nodular mass measured 16 cm x 11 cm x 9 cm and exhibited a grayish-red, grayish-yellow appearance with a rough surface. Upon sectioning, it revealed localized areas of gray-white solid tissue with hemorrhagic, necrotic and crispy areas. Additionally, two nodular masses measuring 1.5-3 cm in diameter were observed in the colonic sulcus. These masses displayed a gray-pink surface and a gray-white section with a firm texture. Furthermore, three mesenteric nodular masses measuring 0.5-1.5 cm in diameter were identified to have similar characteristics of a gray-pink surface, gray-white section, and hard texture.

Microscopically examination revealed the presence of multinucleated tumor giant cells. The tumor cells showed pleomorphic changes arranged in an irregular pattern along with numerous pathological mitotic figures (**Figures 2C–F**).

The pathological diagnosis indicated the presence of giant cell-rich malignant tumor accompanied by necrosis within the pelvis, as well as malignant tumor infiltration featuring calcification and ossification within the fibrous tissue of the colonic groove and mesenteric nodule. It is considered to be a sarcoma-like mural nodule with local sarcoma formation (undifferentiated sarcoma).

**Figure 3:**
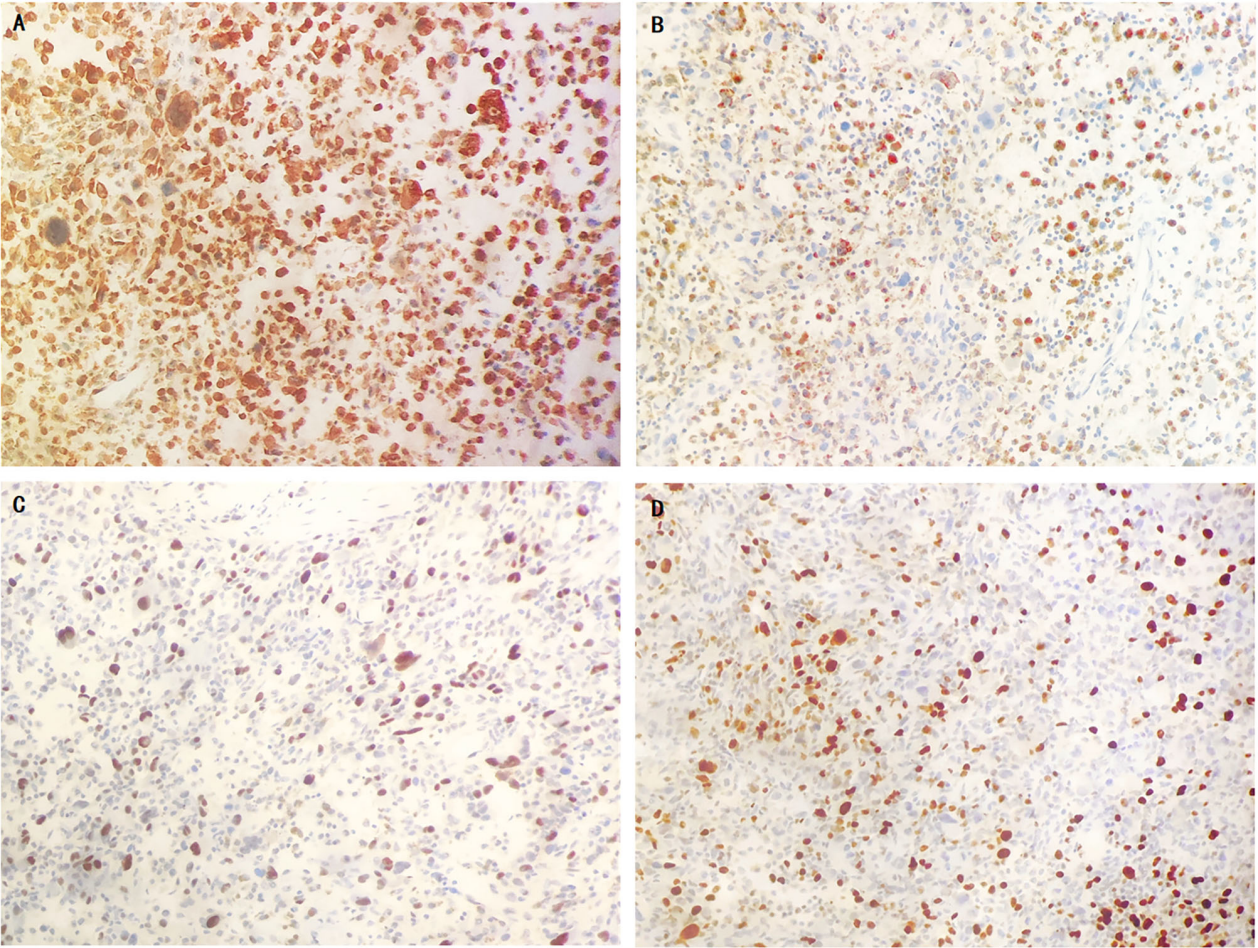
The immunohistochemical results of sarcoma component. tumor cells showed diffusely strongly positive for vimentin **(A)**, partially positive for CD68 **(B)**, 60% positive for P53 **(C)** and Ki67 **(D)**.

Immunohistochemical analysis demonstrated strong positive expression of vimentin (VIM) and CD68 in tumor cells. Tumor cells expressed P53 and Ki-67 at approximately 60%, while cytokeratin (CK), SMA,Des,CK8/18,CK7,Pax-8,WT-1,CK20,ER,PR,CA125,ALK-1,CK5/6(-),CD10(-),CR(-),EMA(-),D2-40,P16were negative (**Figure 3**).

### DNA sequencing

DNA was extracted separately from tissue sections of primary mucinous ovarian carcinoma and recurrence sarcomatous mural nodules using standard laboratory procedures. Next-generation sequencing (NGS) was performed by Geneplus Ltd (Beijing, China), using custom-designed probes covered approximately 1.1 Mb of genomic sequences of cancer-related genes for DNA capture. The 1021-gene panel sequencing was carried out on a Geneplus 2000 Sequencing System (Beijing, China) with 2×100 bp paired-end reads. The case was annotated for point mutations, small insertions and deletions (indels), copy number alterations, microsatellite instability, and tumor mutational burden. Matched blood samples were sequenced as control. Shared pathogenic KRAS alterations (c.35G>T, p.G12V), TP53 alterations(c.817C>T,p.R273C), MLL2(c.13450C>T,p.R4484) and NF1(c.7876A>G,p.S2626G) were detected in the mucinous ovarian carcinoma and sarcomatous mural nodules. Besides, mucinous ovarian carcinoma harbored NLRC3 mutation (c.377G>A,p.R126Q). Sarcomatous mural nodules showed APC(c.3625G>A,p.E1209K) mutation and KRAS copy number alterations (NM_033360.2, 2.0 amplification). All mucinous ovarian tumors and mural nodules were microsatellite stable. Tumor mutational burden is 0.96mutations/megabase in mucinous ovarian tumors and 2.25 mutations/megabase in mural nodules.

## Discussion

From 1979 to 2021, there were only 12 cases of ovarian mucinous tumors with sarcomatous mural nodules (**Table 1**). To our knowledge, this is the first case of SLMNs and undifferentiated sarcoma after treatment of ovarian mucinous cystadenocarcinoma of the ovary with KRAS, TP53, MLL2 and NF1 mutation. We summarize the characteristics of sarcomatous mural nodules and their differentiation from sarcomatoid mural nodules and provide a feasible treatment plan.

Mucinous tumors with mural nodules, such as sarcoma-like mural nodules (SLMNs), sarcomatous mural nodules, or anaplastic carcinoma, usually occur in giant ovarian cystic tumors. Sarcomatous nodules commonly occur in stage I ovarian mucinous tumors in women aged 18 to 65 years. The tumor markers did not increase significantly before the second surgery, which was similar to previously reported tumor markers. In 6 cases with reported tumor markers, serum tumor markers CA125 and CA199 increased slightly or remained normal, and no specific tumor markers of mural nodules were identified.

The prognosis of sarcomatous mural nodules is poor, and the prognosis of sarcoma-like nodules is better. In this case, recurrence and rapid growth were found 4 months after surgery, and the size of the nodule was 16 × 11 × 9 cm at the time of reoperation 8 months later, demonstrating the biological behavior of a sarcoma with rapid growth. Similar to our case, 2 cases of mucinous carcinoma with sarcomatous mural nodules. One patient developed liver metastasis after 36 months, and the other died of renal failure after surgery (1). A stage IIa mucinous carcinoma with sarcomatous mural nodules was reported lung metastasis at 18 months of follow-up (9). In contrast, 7 cases of SLMN reported by J. Prat and R.E. Scully were followed up for 1 to 11 and a half years (mean 7.5 years) with no evidence of recurrence (12). These cases suggest that sarcomatoid mural nodules are not sarcomas and may be a reactive process that does not affect the prognosis of patients (13). However, sarcomas are highly invasive and have a poor prognosis in previous case reports, even in patients with stage Ia ovarian mucinous carcinoma. Stage I primary highly differentiated ovarian mucinous adenocarcinoma has a good prognosis and a high etiology-specific 10-year survival rate (92.7%). In contrast, undifferentiated soft tissue sarcoma has a poor prognosis, with local recurrence rates of 19-31%, metastasis rates of 31-35%, 5-year survival rates of 65-70% and a median survival of approximately 12 months (14, 15). Therefore, the identification of sarcomatous mural nodules is particularly important for the management and prognosis of patients.

Gross morphology and microscopic pathologic diagnosis of mural nodules are essential to identify sarcomatous mural nodules. According to the description of previous cases, sarcomatous nodules are characterized by large volume, obvious hemorrhage and necrosis, indistinct border infiltration, with or without lymphatic vascular invasion, often containing mononuclear or multinucleated malignant tumor cells, giant cells, obvious nuclear atypia and atypical mitosis and inconspicuous inflammatory infiltration (1, 16). The size of the tumor was more than 10 cm, and necrosis in the central area was obvious. In our case, the tumor cells showed polymorphic changes with odd-shaped tumor giant cells and a large number of pathological nuclear divisions. In contrast, SLMNs are predominantly small in size, well demarcated from adjacent mucosal epithelium, and composed of mixed cell groups, including inflammatory cells and osteoclast-like giant cells, which may be prominent around the central hemorrhagic cavity, with significant inflammatory infiltration without vascular infiltration (12, 17). Although morphologic differences have been described, sarcoma and sarcomatoid nodules are not always easy to distinguish morphologically. Both of them have necrosis, obvious nuclear atypia and abundant mitosis (including atypical forms) (13, 17). In this case, atypical vimentin-positive spindle cell and CD68-positive giant cell in the sarcoma component are similar to one case of sarcoma and one case of osteosarcoma nodule (7, 10).Sarcomatoid nodules were sometimes weakly positive for CK (18). Immunohistochemistry of the sarcoma component in this case failed to classify the nodules as any conventional sarcoma because it lacked markers of smooth muscle, skeletal muscle and endothelial differentiation.

On the origin of mucinous cystic tumors of the ovary with mural nodules, SLMNs may be benign reactive changes to intramural bleeding or cyst mucus content, resulting in pseudotumors, such as inflammatory pseudotumors or inflammatory myofibroblastoma elsewhere (17). At present, there are two main theories to explain the origin of sarcomatous mural nodules: (1) The collision theory holds that two tumor types, epithelial cells and mesenchymal cells, collide independently, showing that carcinoma and sarcoma are two independent tumors with polyclonal characteristics. (2) The transformation theory suggests that the sarcoma component is derived from cancer during tumor evolution or the differentiation of primitive stem cells into one cell type, which in turn differentiates into a second cell type (19). In our case, Gene detection results support the transformation theory, because ovarian mucinous carcinoma and sarcomatous nodules had the same KRAS, TP53, NF1 and MLL2 mutations. Consistent with our case, David B. Chapel found a clonal relationship between mural nodules and ovarian mucinous tumors, with KRAS alterations and TP53 mutations in 10 of 13 cases. There was a codon 12 mutation with different nucleotide substitutions of the K-RAS gene in both ovarian invasive mucinous adenocarcinoma and high-grade sarcomatous mural nodules, suggesting that sarcomatous mural nodules may be a dedifferentiated form of mucinous tumors (20). Increased expression of tumor protein p53, and p53 alongside Wilms tumor 1 (WT1) are associated with reduced overall survival and dismal prognosis of carcinomas and carcinosarcomas (21). In contrast, in another case, loss of heterozygosity (LOH) was present at the D18S51 and FGA loci in the adenocarcinoma component, while LOH was present at the D19S433 locus in the sarcoma component, supporting that sarcoma nodules and mucinous tumors may represent the tumor collision phenomenon (9).

Although recurrence of mucinous ovarian cancer presenting with sarcomatous mural nodules has not been reported, recurrence of epithelial tumors with sarcoma components exists. Seven cases of ovarian cancer recurring as carcinosarcoma have been reported in the past (22). In recent years, these tumors are considered to be metaplastic carcinomas. Frederick found that the composition of metastatic lesions changed over time based on pathology results from 26 women diagnosed with ovarian carcinosarcoma. Two cases of epithelial ovarian cancer relapsed as carcinosarcomas, and the incidence of metastatic lesions dominated by sarcomas was three times higher than that of initial surgery (24% vs 8%, respectively), supporting the potential for phenotypic transformation during ovarian cancer progression (23, 24). In our case, all sections from previous biopsies were re-examined, but no sarcomatous component was detected (25). According to the transformation theory and the hypothesis of Wen Wang (8). We suggest that the subepithelial mesenchymal cells react with intramural bleeding or mucinous contents, resulting in the formation of proliferative nodules and enlargement into the SLMN, mesenchymal cells continue to proliferate and differentiate, and part of the SLMN is transformed into true sarcoma.

It is noteworthy that P53, KRAS was expressed in both primary and recurrent sarcomatous tumors. In other cases, p53 was overexpressed in almost all serous ovarian carcinoma and recurrent carcinoma and sarcoma components (24). The most common genetic alterations in ovarian mucinous tumors are somatic KRAS mutations, with an incidence of 50% to 68% in borderline and malignant tumors (15–17). Some studies believe mural nodules in mucinous ovarian tumors do not associated with KRAS and TP53 genetic abnormalities, as the mutation rates of KRAS and TP53 are equally high in both components (11). Recently, it has been suggested that vimentin expression may be associated with the ability of high-grade epithelial tumors to recur as carcinosarcomas or sarcomas. Breast cancers expressing vimentin proteins may originate from progenitor cells with bilinear (glandular and myoepithelial) differentiation potential (26).

A comprehensive genomic analysis has reported that the NF1 gene is one of the most frequently altered genes in soft tissue sarcomas (STS) (27). Studies have demonstrated that individuals with NF1 mutations are predisposed to developing STS (28). *In vitro* experiments, NF1 promotes the initiation and progression of UPS by inhibiting the PI3K-Akt-mTOR-S6 pathway (28). PI3K pathway is frequently upregulated in epithelial ovarian cancers and plays an important role in cell survival. Chemoresistance and preservation of genomic stability, the inhibition of the PI3K may lead to genomic instability and mitotic catastrophe (29). SIFT (30) and Polvphen-2 (31) predicted a deleterious effect of NF1 mutation in this patient, which may be associated with sarcoma development.

Somatic mutations of MLL1 implicated in the pathogenesis of cancer. High mutation rates have been observed in endometrial carcinoma, esophageal sarcomatoid carcinoma, gastric cancer (32). In colon cancer cells lacking MLL2 expression, the target gene expression spectrum of MLL2 revealed its role in promoting transcription of retinoic acid reactive genes such as ASB2. Additionally, MLL2 regulates other transcription factors including NR3C1 and p53. These findings provide insights into the potential mechanism by which MLL2 contributes to cancer progression (33). Specifically, a MLL2 gene c.13450C>T(p.R4484 nonsense mutation) results in an amino acid substitution at position 4484 of the premature termination codon formation, leading to functional impairment or loss of protein expression through nonsense mediated mRNA degradation. The presence of these inactivating mutations suggests that MLL2 functions as a tumor suppressor altering overall gene expression.

Inactivation of the APC gene plays a crucial role in the development and progression of colorectal cancer, while APC gene alterations are rare in sarcomas. One case of primary osteosarcoma reported an APC gene polymorphism (34). Pathogenic mutations in APC lead to aberrant expression of the APC/Axin/Gsk-3 complex or inhibition of the Wnt pathway, resulting in stabilization and accumulation of β-catenin within epithelial cell cytoplasm (35). This allows for migration into the nucleus where it can form a functional transcription factor with TCF/LEF-1 and activate certain oncogenes such as vimentin (35, 36). In this case, missense mutation detection was found in the sarcoma component but not detected in mucinous carcinoma. Software prediction indicated it is a benign mutation. Therefore we do not believe this change drives occurrence of sarcomas. Firstly, it is unlikely that missense mutations alone are responsible for APC gene inactivation. Secondly, compensatory function by axin (another component of the β-catenin/GSK-3β/APC/axin complex) with tumor suppressor activity has already been confirmed (37).

Studies have shown that maximal cytoreduction improves prognosis in patients with advanced ovarian cancer or undifferentiated sarcomas (38). In this case, chemotherapy plus bevacizumab was tried after recurrence, and the effect was not satisfactory, so tumor reduction surgery was performed. After surgery, intraperitoneal hyperthermic perfusion chemotherapy was performed for mucinous cancer components. Intravenous chemotherapy with isocyclophosphamide plus doxorubicin for the sarcoma component. Currently she was treated with amlotinib maintenance therapy according to the Chinese Society of Clinical Oncology (CSCO) 2022 guidelines which recommend amlotinib as a second-line treatment for advanced or unresectable class 1A soft tissue sarcoma. A study used amlotinib as maintenance therapy for soft tissue sarcoma after chemotherapy reported complete relief in one case of undifferentiated pleomorphic sarcoma, achieving an overall response rate (ORR) of 14.3% and disease control rate (DCR) of 81.0% (39). No evidence of recurrence has been observed during the 19-month follow-up period. Additionally, pembrolizumab represents another option for advanced soft tissue sarcomas ineligible for chemotherapy. Targeted therapies aimed at inhibiting the K-RAS signaling pathway may theoretically benefit tumor patients with K-RAS mutations. Promising therapeutic targets have emerged from pathways downstream of Ras activation resulting from NF1 mutations (40).

## Conclusions

In conclusion, ovarian mucinous cystic tumors with mural nodules are a rare and unique type of ovarian tumor that often occurs in giant ovarian cysts. Patients with sarcoma components tend to relapse rapidly and have a poor prognosis. According to previous reports and the genetic analysis of our case, we believe that the sarcoma component may be formed by the transformation of epithelial components and associated with NF1, MLL2 mutations. In clinical practice, doctors should take care to preserve the integrity of cysts during surgery. Pathologists need to take comprehensive samples of mucinous tumors and carefully evaluate them to avoid misdiagnosis and missed diagnosis.

## Data availability statement

The original contributions presented in the study are included in the article/**Supplementary Material**. Further inquiries can be directed to the corresponding author.

## Ethics statement

Written informed consent was obtained from the individual(s) for the publication of any potentially identifiable images or data included in this article. 

## Author contributions

SL: Data curation, Methodology, Project administration, Validation, Writing – original draft, Writing – review & editing. JZ: Methodology, Writing – review & editing, Software, Visualization. NJ: Data curation, Investigation, Writing – review & editing, Conceptualization, Methodology, Visualization. YG: Writing – review & editing, Data curation, Investigation. MH: Investigation, Methodology, Writing – review & editing. XL: Data curation, Visualization, Writing – review & editing. JY: Investigation, Writing – review & editing. XY: Funding acquisition, Project administration, Writing – review & editing.

## Supplementary material

Supplementary Figure 1 Timeline with relevant data from the episode of care.

Supplementary Figure 2 Heatmap (ChiPlot) shows the comparsion of gene mutation frequency between primary mucinous ovarian carcinoma and recurrence sarcomatous mural nodules. KRAS alterations,TP53 alterations, MLL2 and NF1 were detected in the mucinous ovarian carcinoma and sarcomatous mural nodules. Mucinous ovarian carcinoma harbored NLRC3 mutation. Sarcomatous mural nodules showed APC mutation.
