# Supplementary figures and images for: Recurrent mucinous carcinoma with sarcomatoid and sarcomatous mural nodules: a case report and literature review
Source: Front Oncol. 2024 Jun 6;14:1387700. doi: 10.3389/fonc.2024.1387700 (PMC11187075; doi:10.3389/fonc.2024.1387700)

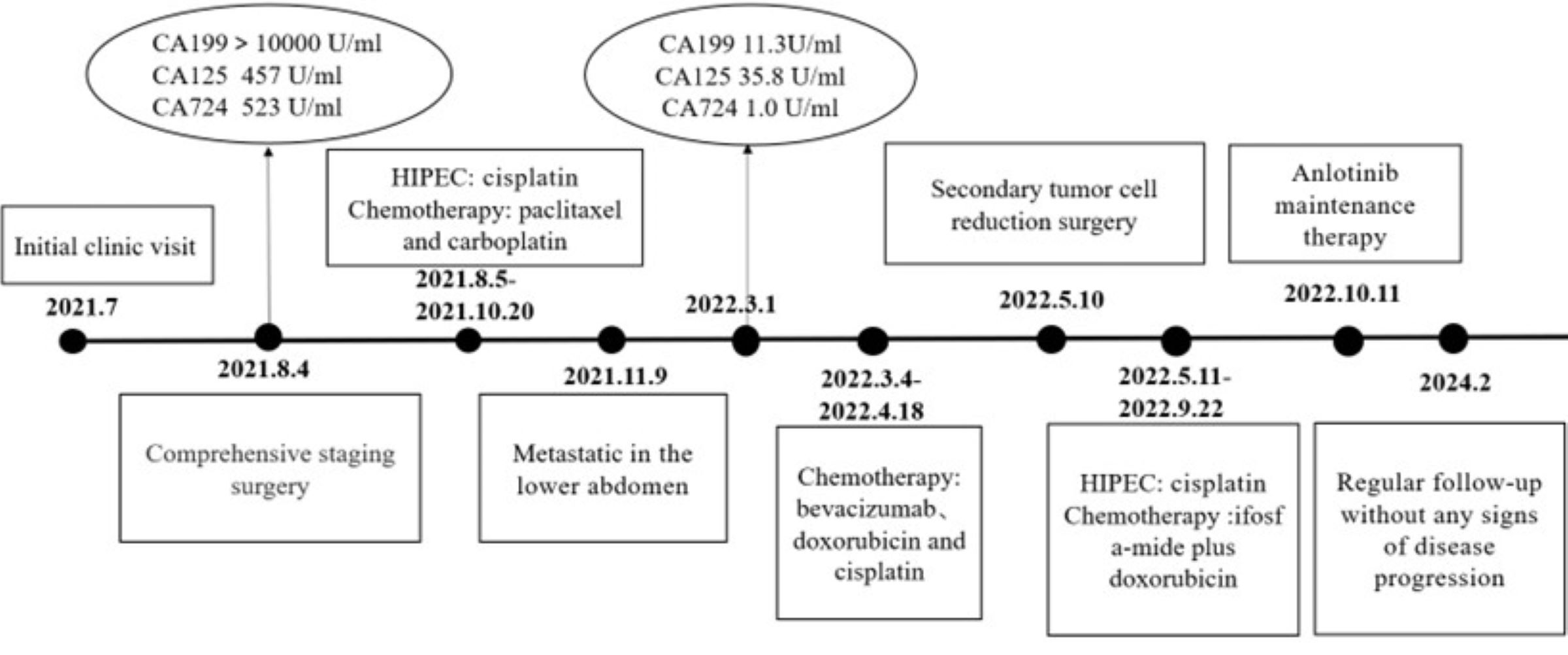

Supplement: Supplementary Figure 1 — Timeline with relevant data from the episode of care. [file Image_1.tif]

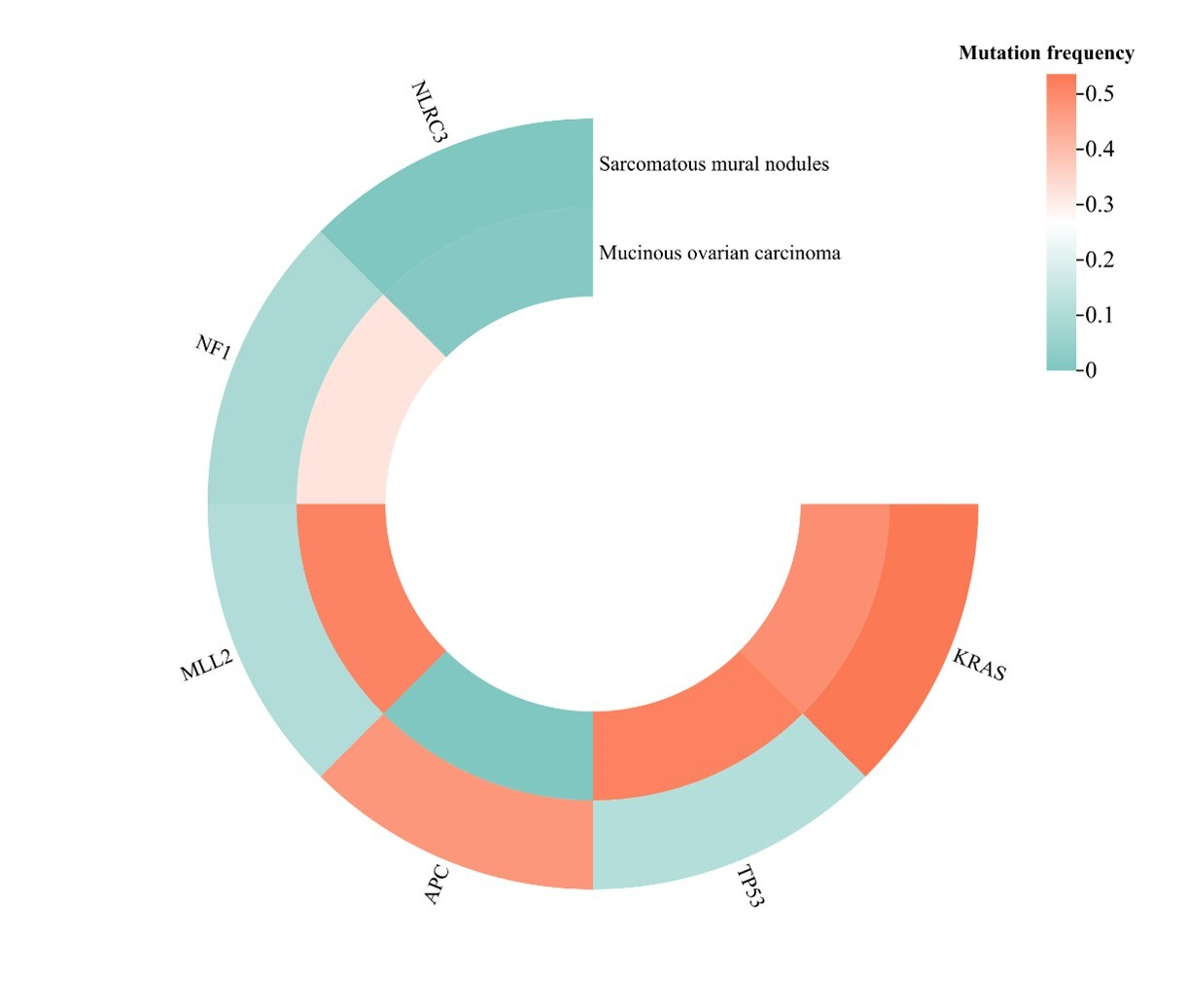

Supplement: Supplementary Figure 2 — Heatmap (ChiPlot) shows the comparsion of gene mutation frequency between primary mucinous ovarian carcinoma and recurrence sarcomatous mural nodules. KRAS alterations,TP53 alterations, MLL2 and NF1 were detected in the mucinous ovarian carcinoma and sarcomatous mural nodules. Mucinous ovarian carcinoma harbored NLRC3 mutation. Sarcomatous mural nodules showed APC mutation. [file Image_2.tif]
